# The role, challenges, and innovations of rifampin in the management of periprosthetic joint infections: a narrative review

**DOI:** 10.1186/s42836-026-00445-z

**Published:** 2026-11-03

**Authors:** Xijiu Zhao, Tiejian Li, Qi Ma, Jiawang Lou, Xu Cai, Changjiao Sun

**Affiliations:** 1 Orthopaedics and Sports Medicine Center, Beijing Tsinghua Changgung Hospital, School of Clinical Medicine, Tsinghua Medicine, Tsinghua University, Beijing, 102218 China; 2 Institute of Orthopaedics and Sports Medicine, Tsinghua Medicine, Tsinghua University, Beijing, 102218 China; 3 Beijing Key Laboratory of Multimodal Intelligent Diagnosis and Therapy for Bone and Joint Injuries & Diseases, Beijing, 102218 China

**Keywords:** Periprosthetic joint infection (PJI), Rifampin, Biofilm, Antimicrobial resistance, Polymethyl methacrylate (PMMA), Microencapsulation, Debridement, antibiotics, and implant retention (DAIR), Antibiotic-loaded bone cement (ALBC), Local drug delivery, Bioengineering

## Abstract

Periprosthetic joint infection (PJI) remains a severe complication due to persistent bacterial biofilms. Rifampin is traditionally valued for its unique biofilm-penetrating properties, yet its clinical role is increasingly viewed as context-dependent. While current evidence supports the efficacy of rifampin in two-stage revision arthroplasty, its application in debridement, antibiotics, and implant retention (DAIR) remains a subject of ongoing debate. Landmark randomized trials once reported superior cure rates for DAIR combined with rifampin, but contemporary large-scale retrospective data show no statistically significant difference in remission rates, often accompanied by a significantly higher incidence of adverse events. Furthermore, the timing of administration is critical; early initiation within five days post-DAIR has been linked to increased treatment failure. These challenges, alongside profound drug-drug interactions, have spurred interest in localized delivery innovations like microencapsulation and nanoparticles. While these bioengineering approaches show preclinical promise in overcoming PMMA chemical incompatibility, they remain at the proof-of-concept stage. Consequently, rifampin should be employed as a targeted adjunct rather than a universal standard, requiring careful consideration of surgical strategy, patient tolerance, and optimal timing.

**Supplementary Information:**

The online version contains supplementary material available at https://doi.org/10.1186/s42836-026-00445-z.

## Introduction

The increasing number of total joint arthroplasties being performed throughout the world continues to be mirrored by an increase in periprosthetic joint infections (PJI). PJI is still one of the most severe complications in orthopedic surgery and poses health-related (both physical and psychological) challenges to patients, leaving a heavy burden on society and a significant financial impact on healthcare systems [1–3]. PJI is inherently difficult to manage due to the presence of bacteria localized within biofilms formed on the abiotic surfaces of implanted prostheses.

Biofilms are highly complex and structured communities of bacteria, and sometimes fungi, surrounded by a self-produced enveloping matrix of extracellular polymeric substance (EPS). In these deeper layers of this matrix, bacteria can also go into a metabolically dormant state. Consequently, conventional antimicrobial agents that specifically act on actively dividing cells, such as cell-wall synthesis inhibitors (e.g., β-lactams), lose their bactericidal activity. Such organisms exhibit what is known as antibiotic tolerance, whereby even extended courses of traditional intravenous antibiotics may not achieve sterilization of the infection and can also lead to high rates of relapse once therapy has ended [4].

Surgical source control, from Debridement, antibiotics, and retention (DAIR) to one-stage or two-stage revision arthroplasty, should be administered in conjunction with “biofilm-active” antibiotics to effectively eradicate biofilm-associated infections [5]. Of the few antibiotics that can penetrate biofilms, rifampin is unique. It possesses excellent biofilm penetration capabilities and has been demonstrated to be effective against all forms of Staphylococcus involved in the pathogenesis of PJI, including intracellular, planktonic, and sessile forms [6]. Rifampin, unlike other single agents such as vancomycin, linezolid (LZD), and trimethoprim-sulfamethoxazole (TMP-SMX), is capable of reducing bacteria in both the bone and on the surface of the implant simultaneously [7]. This core element in improving the failure rate of staphylococcal PJIs has been consistently established by multicenter clinical trials and long-term observational studies [8–10].

Rifampin is theoretically and clinically superior but has complications in its real-world application. The need for rifampin to be used only in combination therapy to prevent rapid resistance [7, 11, 12], profound drug-drug interactions (DDI) due to cytochrome P450 induction, and high rates of gastrointestinal and hepatic adverse events often leading to premature treatment termination [13–16]. Additionally, sequestration of rifampin in local bone cement spacers—an ideal approach to avoid systemic toxicity—has been challenged by complex chemical interactions that effectively destabilize the structure of the cement [17].

Rifampin has therefore attracted considerable interest as a biofilm-active agent in the management of staphylococcal PJI, but its clinical use is limited by rapid resistance development, substantial drug–drug interactions, adverse events, and the challenges associated with local delivery through antibiotic-loaded bone cement. In particular, the clinical benefit of rifampin may vary according to the pathogen, surgical strategy, timing of initiation, and companion antibiotic.

This narrative review summarizes the current clinical evidence regarding rifampin use in PJI, with particular emphasis on its efficacy in different surgical settings, treatment-related adverse events and drug interactions, antimicrobial resistance, timing of initiation, and selection of companion antibiotics. We also review emerging bioengineering strategies designed to overcome the limitations of local rifampin delivery, including microencapsulation and nanoparticle-based approaches.

### Literature search and selection

To ensure a thorough and unbiased synthesis of the current evidence regarding rifampin’s role in managing periprosthetic joint infections (PJI), A literature search was conducted to identify relevant studies addressing the clinical use, limitations, challenges, and emerging innovations of rifampin in the management of PJI. The search was conducted across multiple electronic databases, including PubMed, Web of Science, Cochrane Library, Embase, Scopus, ClinicalTrials.gov, as well as major Chinese databases (China National Knowledge Infrastructure (CNKI), Wanfang, China Biology Medicine Disc (CBM), and China Science and Technology Journal (CSTD)). The articles were published from their inception through August, 2026. The detailed search algorithms for each database, including Boolean operators and search terms, are provided in Supplementary Data 1.

**Fig. 1:**
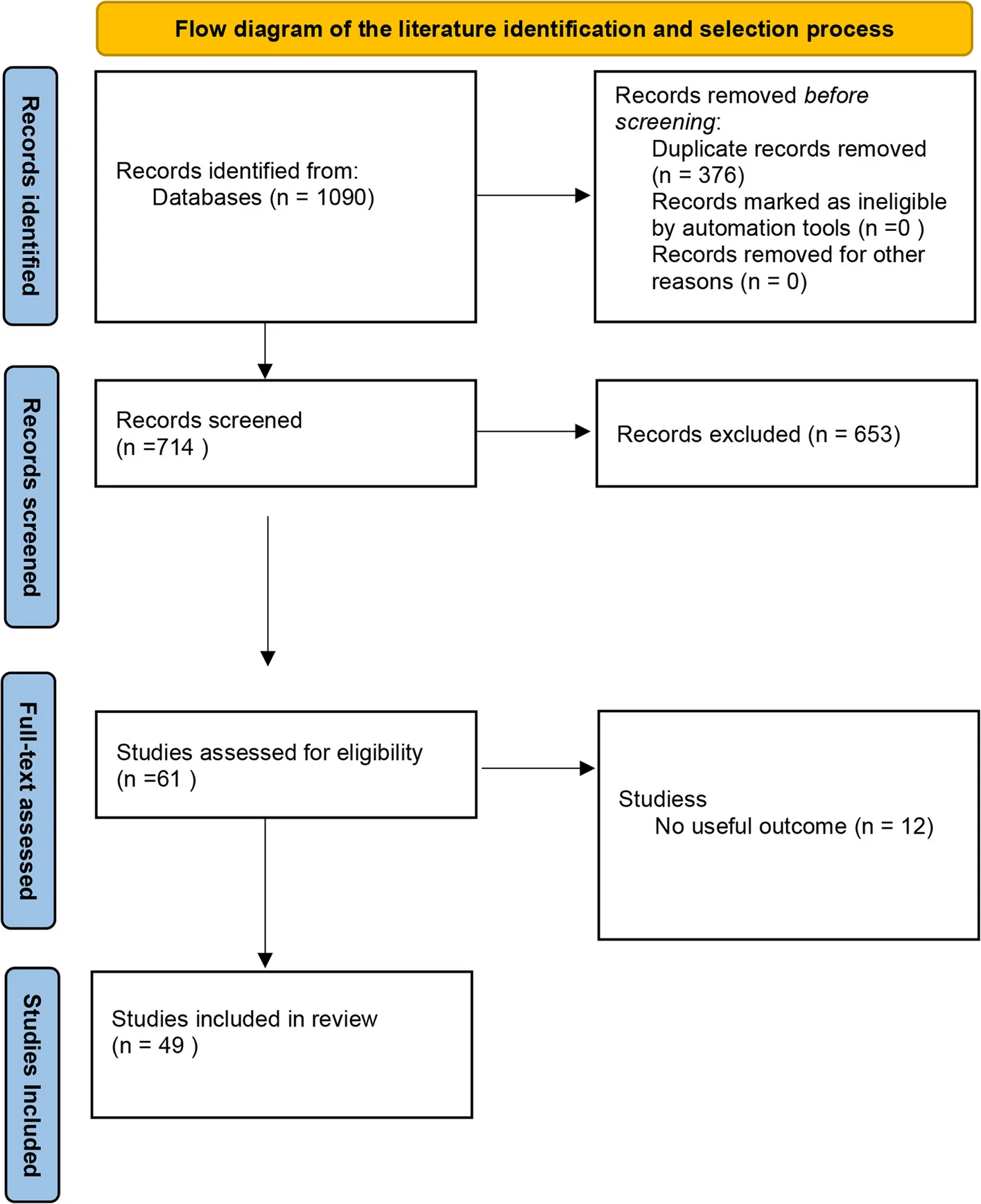
Flow diagram of the literature identification and selection process

The selection of literature was guided by the objective of providing an overview of rifampin’s clinical application and bioengineering innovations in total joint arthroplasty. We included peer-reviewed original research, systematic reviews, and studies that focused on PJI management using rifampin. Exclusion criteria were applied to maintain the review’s focus, primarily omitting studies where rifampin was used for non-prosthetic-related infections. Figure 1 presents a flow diagram of the literature identification and selection process. The initial database search yielded 1090 records. After importing these records into EndNote reference management software, 376 duplicate records were removed. Of the remaining 714 unique articles, 653 were excluded after title and abstract review. The remaining 61 full-text articles were assessed. 12 studies were excluded because of no useful outcomes. Finally, 49 full-text articles were included in the narrative review. In addition, a limited number of relevant background references (e.g., on biofilm biology, antimicrobial resistance, and pharmacology) were cited to provide context but were not part of the formal literature search summarized in Fig. 1.

### Pharmacology, biofilm dynamics, and mechanism of action

#### The microenvironment of biofilm and antibiotic tolerance

PJI is characterized by bacterial biofilm formation on prosthetic surfaces. Within the extracellular polymeric substance (EPS) matrix, bacteria may adopt metabolically dormant states that exhibit increased tolerance to conventional antimicrobial therapy [18, 19]. Biofilm-associated bacteria may therefore require substantially higher antimicrobial concentrations for eradication than planktonic organisms [20]. These features provide an important rationale for the use of antibiotics with biofilm activity, including rifampin, in selected staphylococcal PJIs [21].

#### Rifampin mechanism of action

Rifampin is a broad-spectrum macrocyclic antibiotic originally designed for the treatment of tuberculosis; its bactericidal effect stems from binding to the β-subunit of bacterial DNA-dependent RNA polymerase, which is encoded by the rpoB gene [6]. This prevents the initiation of RNA transcription—a process that is crucial for cellular viability and is required even in semi-dormant bacterial states. Consequently, rifampin preserves its bactericidal activity against slowly growing and non-dividing bacteria embedded deep within the biofilm [6, 12].

From a pharmacokinetic standpoint, rifampin is highly lipophilic with an impressive volume of distribution (0.8 to 1.6 L/kg) and excellent tissue penetration, including into bone, synovial fluid, and the EPS matrix of staphylococcal biofilms [12]. Oral bioavailability is very high (60%–80%), enabling easy conversion from intravenous to oral treatment [12]. This issue is of special importance for the prolonged outpatient treatment commonly needed in cases of PJI.

### Clinical efficacy of systemic rifampin for PJI

#### Rifampin in staphylococcal PJI

Staphylococcus species, especially Staphylococcus aureus (e.g., methicillin-resistant S. aureus, MRSA) and coagulase-negative staphylococci (e.g., Staphylococcus epidermidis), predominated in the PJI microbiome [22]. The gold standard for the treatment of staphylococcal PJIs is currently considered rifampin-combination therapy according to the Infectious Diseases Society of America (IDSA) guidelines [10] and the International Consensus Meeting on Orthopaedic Infection [23]. In particular, the addition of rifampin plus a companion antibiotic, most commonly with fluoroquinolones, has been demonstrated to substantially improve clinical cure rates [8, 9, 24]. A pooled analysis of clinical evidence showed an OR of 2.68 for rifampin combination regimens compared to monotherapies or other combinations [24].

In selected chronic PJI treated with two-stage revision arthroplasty, rifampin in combination therapies had a significantly better treatment outcome. An international, multicenter observational cohort study involving 375 cases of staphylococcal PJI (124 1-stage, 251 2-stage) evaluated the use of rifampin in revision surgery. In the setting of chronic PJIs treated with two-stage revision, the failure rate for the rifampin-treated cohort was significantly lower than that in the non-rifampin group (15% vs 35.5%; *p* = 0.005) [8].

However, rifampin in the setting of debridement, antibiotics, and implant retention (DAIR) is a subject of ongoing debate. Providing Level I/II clinical evidence, the landmark randomized controlled trials, such as the study by Zimmerli et al. in 1998, reported a 100% cure rate with ciprofloxacin-rifampin vs. 58% cured with ciprofloxacin alone [25]. But more recent data tell a different, more complex story. More recent Level III observational data from the largest retrospective cohort study involving 227 patients with staphylococcal PJI who underwent DAIR or revision surgery and found no statistically significant difference in remission rates (62/79 [78.48%] vs. 100/148 [67.57%], *p* = 0.083), while adverse events were significantly more frequent in the rifampin group (31.65% vs. 8.78%, *p* < 0.001) [14]. Importantly, systematic reviews and meta-analyses have also produced somewhat conflicting findings. A systematic review and meta-analysis evaluating staphylococcal PJI treated with DAIR found only a modest association between rifampin use and treatment success (pooled RR 1.10, 95% CI 1.00–1.22), and the authors emphasized the potential influence of confounding, selection bias, and publication bias [26]. In contrast, another systematic review and meta-analysis reported a higher likelihood of therapeutic success with rifampin-containing regimens in staphylococcal PJI treated with DAIR (OR 2.49, 95% CI 1.93–3.23); however, when the analysis was restricted to randomized controlled trials, the benefit was no longer statistically significant (RR 1.27, 95% CI 0.79–2.04) [27]. Recent consensus and expert recommendations, including the 2025 International Consensus Meeting (ICM) [4], continue to recognize rifampin as an important component of biofilm-active therapy for selected staphylococcal PJI, while emphasizing the importance of appropriate patient selection, companion antibiotics, timing of administration, and consideration of drug interactions and adverse effects [4]. To provide a comprehensive perspective on the ongoing debates regarding optimal surgical strategies (DAIR vs. exchange arthroplasty) and the impact of antimicrobial resistance, the key clinical studies evaluating the efficacy of systemic rifampin in staphylococcal PJIs are summarized in Table 1. Collectively, these data suggest that rifampin may provide clinical benefit in selected patients undergoing DAIR, but its magnitude of benefit remains uncertain and may depend on the pathogen, surgical strategy, timing of administration, companion antibiotic, modular exchange. and patient-specific factors.

**Table 1:** Summary of key clinical studies evaluating rifampin efficacy in staphylococcal PJI

Author & Year	Study Design & Sample Size	Pathogen Type	Surgical Strategy	Key Findings & Clinical Outcomes
Zimmerli et al., 1998 [25]	RCT (*n* = 33)	*Staphylococcus aureus* (MSSA)	DAIR	(18 ciprofloxacin-rifampin vs. 15 ciprofloxacin-placebo). The cure rate was 100% (12/12) in the ciprofloxacin-rifampin group vs. 58% (7/12) in the placebo group (*p* = 0.02) at ~ 34 months follow-up. 9 patients dropped out (6 due to adverse events)
Kramer et al., 2024 [8]	Multicenter Observational Cohort (*n* = 375)	*Staphylococcal* spp.	1-stage & 2-stage exchange	(124 1-stage, 251 2-stage). Overall, rifampicin showed no significant benefit (*p* = 0.051). However, in the 2-stage exchange subgroup, rifampicin significantly reduced failure (15% vs 35.5%, *p* = 0.005). Rifampicin (OR 0.36) and antibiotic holiday > 2 weeks (OR 0.19) were independent predictors of success
Zhang et al., 2025 [14]	Retrospective Cohort (*n* = 227)	*Staphylococcal* spp.	DAIR, 1-stage, 2-stage	(79 Rif vs 148 Non-Rif). No significant difference in remission rate (62/79 [78.48%] vs. 100/148 [67.57%], *p* = 0.083) or survival (*p* = 0.509) between groups. No benefits were found in DAIR, 1-stage, or 2-stage subgroups. However, adverse events were significantly higher in the rifampin group (31.65% vs 8.78%, *p* < 0.001)
Eriksson et al., 2023 [40]	Retrospective Cohort (*n* = 81)	*Rifampin-resistant Staphylococcal* spp.	DAIR (Early PJI)	Compared outcomes of Rifampicin-resistant (Rif-R) vs. Rifampicin-sensitive (Rif-S) staphylococci in DAIR. Relapse rates were significantly higher in the Rif-R group after 1 st DAIR (80% vs. 51%, *p* = 0.04) and 2nd DAIR (70% vs. 33%, *p* = 0.03). Rif-R increased relapse risk by 1.9- to 4.1-fold
Grünwald et al., 2025 [43]	Retrospective Cohort (*n* = 212)	*Staphylococcal* spp.	2-stage exchange	(60.8% THA, 39.2% TKA) undergoing 2-stage revision. Rifampicin combination therapy started on Day 2 post-debridement. At a mean 55.4-month follow-up, acquired rifampicin resistance was rare: 0.9% at spacer explantation and 1.4% after reimplantation. Overall reinfection rate: 11.8% (25/212)
Lazarinis et al.,2023 [39]	Retrospective Cohort (*n* = 238)	*Staphylococcal* spp.	DAIR, 1-stage, 2-stage	2-year infection-free survival was significantly lower in the Rif-resistant group (78.6%) than the Rif-sensitive group (90.0%). Rif resistance was associated with a 4.2-fold increased risk of treatment failure
Lora-Tamayo et al., 2017 [30]	Multinational Study (15 countries/52 centers) (*n* = 462)	*Streptococcus* spp.	DAIR	Overall failure rate was 42.1%. Success was independently associated with early use of rifampin (HR 0.98 per day in the first 30 days), exchange of removable components (HR 0.60), and long-term (≥ 21 days) β-lactam therapy. The combination of β-lactams + rifampin was more protective (HR 0.34) than β-lactam monotherapy (HR 0.48)
Kusejko et al., 2020 [31]	Multinational Study (9 countries/18 centers) (*n* = 187)	*Cutibacterium* spp.	DAIR, 1-stage, 2-stage	Adding rifampin showed a trend toward benefit but was not statistically significant for failure (aHR 0.5, *p* = 0.07) or relapse (*p* = 0.10). Significant predictors of failure were DAIR (aHR 2.15, *p* = 0.03) and antibiotic treatment ≤ 6 weeks (*p* = 0.0002)

#### Rifampin in the setting of non-staphylococcal PJI

Although the major indication for rifampin is in staphylococcal infections, it has also shown potential in vitro efficacy against other biofilm-forming microorganisms such as *Streptococcus* species and *Cutibacterium* acnes (formerly known as *Propionibacterium acnes*) [28, 29]. A large international multicenter study by Lora-Tamayo et al. [30] assessed 462 patients treated solely with DAIR. The overall failure rate was high, at 42.1%. Multivariate analyses further demonstrated that rifampin given earlier (as judged by the cumulative days of treatment in the first 30 days after debridement) was independently associated with clinical success. Furthermore, prolonged therapy (≥ 21 days) with β-lactams, either in combination with rifampin or as monotherapy, alongside the exchange of removable modular components, significantly improved outcomes. This suggests a tangible benefit of early rifampin adjunct therapy in streptococcal PJI following DAIR. Conversely, the benefit of rifampin in *Cutibacterium* PJIs appears less definitive and is often overshadowed by surgical variables. A multicenter study of 187 *Cutibacterium* PJIs demonstrated that adding rifampin provided only tentative, non-statistically significant reductions in treatment failure and relapses. Instead, clinical success was primarily dictated by surgical strategy and treatment duration: prolonged antibiotic therapy (> 6 weeks) significantly reduced failure risk [31].

Overall, the clinical utility of rifampin in non-staphylococcal PJI appears to be pathogen- and treatment-strategy dependent. Evidence for a potential benefit is more supportive in streptococcal PJI managed with DAIR, whereas its benefit in *Cutibacterium* PJI remains uncertain. Rifampin should therefore not be routinely extrapolated to all non-staphylococcal PJIs and should be considered according to the causative organism and overall treatment strategy. The key findings from relevant clinical studies are compiled in Table 1.

### The adverse events and DDIs

While rifampin therapy showed potential efficacy, its clinical utilization is frequently diminished due to marked tolerability concerns. The drug is well-recognized for causing a broad spectrum of adverse reactions.

#### Gastrointestinal and hepatic toxicity

There is considerable clinical evidence of the prevalence of rifampin therapy side effects. In patients with PJI, as high as 91% develop disturbances of their gastrointestinal tract, such as nausea, vomiting, and significant abdominal discomfort, often requiring dose reductions in about 29% of these cases [13]. Another study, including 227 patients, reported that systemic rifampin was associated with more than a fourfold higher rate of overall adverse events compared to regimens not containing rifampin [14].

Hepatotoxicity represents another critical concern. Rifampin may cause transient increases in liver transaminases and, rarely, clinical hepatitis [16]. This makes it essential to perform stringent and continuous monitoring of liver function tests over the treatment period, which can last from 3 to 6 months.

#### Significant DDI

Rifampin is one of the most potent inducers of the hepatic cytochrome P450 enzyme system–especially CYP3A4, CYP2C9, and CYP2C19. This induction increases the metabolism of many co-administered drugs and poses a major challenge in the arena of PJI. About 18.4% of patients with PJI are absolutely contraindicated for systemic rifampin, due to severe and/or unmanageable drug-drug interactions [15]. Even with patients who fulfill the IDSA definition for rifampin therapy, almost 50% are unable to initiate prescribing due to pre-existing hepatic or renal impairment or medications that cannot be used concomitantly (e.g., oral anticoagulants like warfarin or direct oral anticoagulants, which are common in orthopedic populations) [16].

In addition, rifampin speeds the clearance of traditional companion antibiotics. It is well known to drastically reduce the plasma concentrations of doxycycline, TMP-SMX, clindamycin, and fusidic acid [32]. Thus, clinicians are confronted with a challenging therapeutic landscape—trying to use rifampin effectively to treat biofilm infections while also lowering the serum levels of co-administered antibiotics and increasing the risk of both systemic treatment failure and resistance development.

The clinical implications of discontinuing rifampin therapy are serious. Similar studies from the Mayo Clinic show that rifampin treatment completion is critically important because interruption of rifampin administration due to intolerance or drug-drug interactions results in a 3.5-fold increase in PJI treatment failure rate [9].

For the 18.4% of patients with rifampin contraindications, Rifabutin and rifapentine are viable substitutes. Rifabutin exhibits a significantly lower DDI rate (3.4% vs. 18.4%), facilitating its use with oral anticoagulants [15].In vitro assays suggest rifabutin may even surpass rifampin in anti-biofilm efficacy, showing lower minimum biofilm eradication concentrations (MBECs) against staphylococci [33]. While preclinical animal models confirm rifabutin and rifapentine are as effective as rifampin against MRSA foreign-body infections [34], their activity against streptococci and enterococci biofilms remains limited [35]. Consequently, these derivatives serve as targeted alternatives for staphylococcal PJI when rifampin-related toxicity or interactions necessitate therapy substitution, though their role in non-staphylococcal infections is less robust.

#### Drug-induced fever

Rifampin-induced fever is an uncommon but clinically important adverse event because it may mimic persistent or recurrent infection. Case reports have described high fever occurring during rifampin therapy that resolved after drug withdrawal, with recurrence on rechallenge in some cases [36, 37]. Awareness of this adverse reaction may help avoid unnecessary investigations or inappropriate escalation of antimicrobial therapy.

Navigating the administration of systemic rifampin requires constant clinical vigilance to balance its anti-biofilm benefits against its profound toxicities. To assist clinicians in managing these multifaceted challenges, the major drug-drug interactions, systemic adverse events, and recommended clinical management strategies are consolidated and outlined in Table 2.

**Table 2:** Major drug-drug interactions (DDIs) and adverse events associated with systemic rifampin in PJI management

Category	Interacting Agent/Issue	Mechanism	Clinical Consequence in PJI	Management/Recommendation
Companion Antibiotics	Doxycycline, Linezolid, TMP-SMX, Clindamycin, Fusidic Acid	Potent induction of hepatic Cytochrome P450 (CYP3A4, CYP2C9)	Accelerated metabolism leads to sub-therapeutic plasma levels of companion drugs, risking treatment failure	Monitor drug levels if possible; consider alternative regimens; strictly avoid monotherapy
Systemic Medications	Warfarin, Direct Oral Anticoagulants (DOACs)	Induction of CYP450 and P-glycoprotein efflux transporters	Drastically reduced anticoagulant efficacy, increasing risk of deep vein thrombosis (DVT) or pulmonary embolism (PE)	Switch to low-molecular-weight heparin (LMWH) or frequently monitor INR (for warfarin); avoid DOACs
Systemic Toxicity	Gastrointestinal (GI) Tract	Direct mucosal irritation	Nausea, vomiting, abdominal pain; leads to forced dose reduction in ~ 29% of patients	Administer on an empty stomach if tolerated, or divide doses. Stop therapy if severe
Hepatic Toxicity	Liver enzymes	Hepatocellular injury	Transient transaminitis to clinical hepatitis	Baseline and bi-weekly liver function tests (LFTs). Discontinue if AST/ALT > 5 × upper limit of normal
Misdiagnosis Risk	Drug-Induced Fever	Hypersensitivity reaction	High fever (up to 40 °C) without inflammatory symptoms	Misdiagnosed as PJI relapse. Perform a re-challenge test; discontinue rifampin to resolve fever

### The threat of rifampin resistance

Since rifampin binds to and specifically inhibits a single bacterial enzyme, high-level resistance can develop quickly from point mutations in the rpoB gene that confer drug resistance [38]. As a result, rifampin should never be used as monotherapy.

The rise of Rifampin-resistant Staphylococci is an important clinical challenge. In a cohort study with data from a single center in Sweden, including 238 patients with staphylococcal PJIs, rifampin-resistant strains were observed in 17% of the cases. Notably, 2-year infection-free survival rates fell from 90.0% in the rifampin-sensitive cohort to only 78.6% among those infected with resistant strains. For patients with rifampin-resistant bacteria, the Hazard ratio for treatment failure was 4.2 [39].

The implications of resistance are particularly limiting in the setting of DAIR procedures. Eriksson et al. studied 81 patients with early PJIs treated with DAIR. The reinfection rate after the initial DAIR procedure in patients infected with rifampin-resistant staphylococci reached a substantial 80% (after the first DAIR procedure) and 70% (relapse after re-DAIR). Compared to patients infected with rifampin-sensitive strains, the adjusted odds of infection relapse in patients with rifampin-resistant strains were approximately four times higher after two DAIR procedures. These findings suggest that rifampin resistance may be associated with poorer outcomes in patients undergoing DAIR. [40].

### Timing of rifampin initiation to prevent resistance

The optimal timing of rifampin initiation remains controversial and appears to depend on the surgical strategy. In patients undergoing DAIR, early initiation may be associated with poorer outcomes. In a multicenter observational study of 669 patients with acute staphylococcal PJI treated with DAIR, rifampin use was associated with treatment success overall, but initiation within five days after debridement was independently associated with treatment failure (OR 1.96, 95% CI 1.08–3.65) [41]. The timing of surgical source control may also be critical: DAIR performed within one week of symptom onset was associated with significantly higher infection eradication rates, indicating that timely surgery is a prerequisite for adjunctive rifampin therapy to be effective [42].In contrast, early rifampin initiation may be feasible after two-stage exchange, in which the infected prosthetic material has been removed. In a retrospective study of 212 patients undergoing two-stage revision, rifampin-based therapy initiated by postoperative day 2 was associated with low rates of acquired rifampin resistance during follow-up [43]. These findings suggest that the timing of rifampin initiation should be individualized according to the surgical strategy, extent of source control, and risk of resistance.

### Local delivery innovations

#### The polymethyl methacrylate (PMMA)

**Fig. 2:**
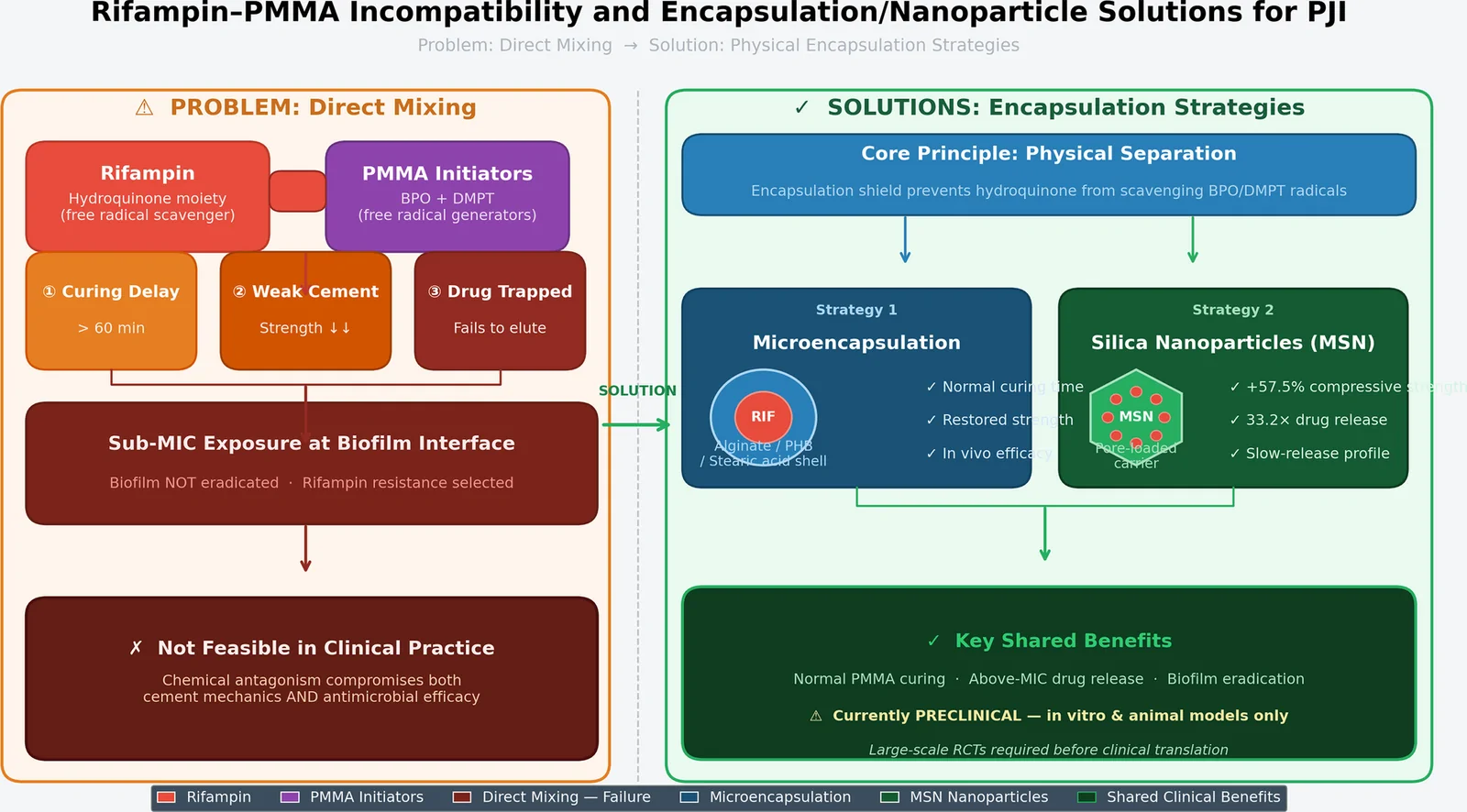
Schematic illustration detailing the molecular interference of rifampin with PMMA polymerization (left) and the protective role of nanostructured carriers in preserving cement integrity (right)

Due to the severe systemic toxicities, high rates of intolerance, and DDIs attributed to oral or intravenous rifampin, it has been proposed that a solution for PJI would be delivery of rifampin directly into the joint space by use of antibiotic-loaded bone cement (ALBC). Such a strategy would allow high concentrations of rifampin to be delivered directly at the biofilm interface, while minimizing systemic side effects [44]. However, direct incorporation of rifampin into PMMA bone cement is not feasible in clinical practice. PMMA polymerization is an exothermic radical chain reaction that begins by means of the use of an initiator, dibenzoyl peroxide (BPO), and an accelerator, dimethyl-p-toluidine (DMPT). Rifampin contains a hydroquinone (benzene-1,4-diol) moiety. This hydroquinone group will act as an excellent free radical scavenger during the PMMA mixing stages. Rather, rifampin competes strongly with the BPO/DMPT initiators, successfully terminating the free-radical polymerization chain [45]. The chemical conflict causes a dramatic delay in the setting time of the cement (> 1 h) and also causes incomplete polymerization, resulting in much lower mechanical compressive strength of the spacer and pathological surface porosity [17]. The chemical antagonism between rifampin’s hydroquinone moiety and the free-radical chain reaction of PMMA, which leads to compromised mechanical strength, is schematically illustrated in the left panel of Fig. 2

Initial attempts to reduce this problem involved substantially lowering the quantity of rifampin incorporated into cement. However, Likine et al. showed that even a dose reduced to 300 mg was still associated with a 20-min delay in curing time [46]. Laperche et al. also showed that even 100 mg of rifampin to 40 g of bone cement reduced the mechanical strength [47]. More concerning, Han et al. showed that mixing rifampin directly into the cement matrix essentially traps it and overwhelmingly fails to elute. This leads to local concentrations of rifampin below the minimum inhibitory concentration (sub-MIC) that not only do not kill the biofilm but also select for mutant strains with increased rifampin resistance [48].

#### Advanced bioengineering approaches: microencapsulation and nanoparticles

The local delivery strategies discussed in this section remain at the preclinical or proof-of-concept stage and should not be considered established clinical treatments. Microencapsulation and nanoparticle-based approaches have been investigated to physically separate rifampin from the reactive components of PMMA, thereby reducing interference with polymerization while improving drug release [17, 49, 50]. Microencapsulated rifampin formulations have been shown to preserve PMMA curing characteristics and mechanical properties, while animal studies have demonstrated sustained local drug release and antimicrobial activity [17, 50]. Mesoporous silica nanoparticles have also been investigated as rifampin carriers and may improve both PMMA mechanical properties and drug release compared with conventional direct mixing [49]. A comprehensive comparative analysis of traditional direct mixing versus these cutting-edge microencapsulation and nanoparticle strategies is presented in Table 3, highlighting their respective impacts on PMMA curing times, mechanical strength, and in vivo antimicrobial efficacy. To circumvent this molecular interference, bioengineering approaches utilize physical sequestration—depicted in the right panel of Fig. 2—to protect the polymerization process while enabling sustained drug elution. While these preclinical findings suggest a promising direction for overcoming the limitations of local rifampin delivery, they represent early proof-of-concept data. Critical challenges such as industrial-scale manufacturing, maintaining structural stability during sterilization, and ensuring long-term in vivo safety must be rigorously addressed. Future research should prioritize validation in large-animal models and safety-focused clinical trials to bridge the current gap between laboratory innovation and standardized orthopedic practice.

**Table 3:** Comparison of local rifampin delivery strategies in PMMA bone cement

Delivery Strategy	Interaction with PMMA Polymerization	Setting/Curing Time	Mechanical Compressive Strength	Drug Elution Profile & In Vivo Efficacy
Direct Mixing (Standard ALBC)	**Severe Conflict:** Hydroquinone moiety in rifampin scavenges BPO/DMPT free radicals	**Severely Delayed:** Often extended > 1 h, clinically impractical	**Significantly Compromised:** Creates pathological surface porosity; structure is brittle	**Poor:** Rifampin gets trapped in the matrix; sub-MIC elution promotes bacterial resistance
Microencapsulation (Alginate/Stearic Acid)	**Protected:** Physical barrier prevents chemical interference with free radicals	**Normal:** Curing time restored to standard PMMA limits (approx. 10–15 min)	**Preserved:** Mechanical strength meets clinical requirements for joint spacers	**Excellent:** Sustained release above MIC; eradicates *S. aureus* in bone/tissue in rabbit models without systemic toxicity
Mesoporous Silica Nanoparticles (MSNs)	**Protected & Enhanced:** Silica nanoparticles shield rifampin and act as reinforcing fillers in PMMA	**Normal:** No interference with the exothermic radical chain reaction	**Significantly Increased:** Enhances compressive strength of PMMA by up to 57.5%	**Superior:** Nanoporous structure provides a 33.2-fold increase in cumulative drug release rate over time

#### Selection of companion antibiotics

The success of rifampin therapy is fundamentally tied to the selection of an appropriate companion antibiotic to prevent the rapid emergence of resistance. While fluoroquinolones (e.g., levofloxacin, moxifloxacin) remain the most evidence-supported partners due to their high oral bioavailability and bone penetration, global emergence of fluoroquinolone resistance represents a major hurdle; in some cohorts, ciprofloxacin-resistant MRSA strains have been observed at rates of 66.7% to 100% [51]. A recent comprehensive meta-analysis of 27 studies [52]involving various agents—including fluoroquinolones, lincomycins (clindamycin), penicillins, linezolid, cotrimoxazole, teicoplanin, and tetracyclines—revealed that a statistically significant improvement in cure rates was only consistently observed when rifampin was paired with fluoroquinolones This disparity is largely explained by pharmacokinetic interference: rifampin’s potent induction of the cytochrome P450 system significantly reduces the plasma concentrations of linezolid, clindamycin, and cotrimoxazole, whereas fluoroquinolone levels remain relatively stable. Although the LZD-rifampin combination has demonstrated superior clearance in quinolone-resistant MRSA models [7], these drug-drug interactions must be carefully managed. Also, clinicians must interpret these pooled data with caution due to study heterogeneity, as this meta-analysis aggregates PJI with spinal and fracture-related infections, which may not fully reflect the unique biofilm dynamics of prosthetic joints.

## Conclusion and future directions

Rifampin remains an important component of the management of selected staphylococcal PJIs, particularly when used as a targeted adjunct in combination with appropriate companion antibiotics and surgical source control. However, its clinical use is context-dependent and must be carefully balanced against the risks of antimicrobial resistance, drug-drug interactions, adverse events, and treatment intolerance. In particular, the available evidence does not support rifampin as a universal standard for all staphylococcal PJIs, and its potential benefits may vary according to the surgical strategy, timing of initiation, pathogen susceptibility, companion antibiotic, and patient-specific factors.

To address the limitations of systemic rifampin therapy, localized delivery systems based on microencapsulation and mesoporous silica nanoparticles have shown preclinical promise in overcoming the chemical incompatibility between rifampin and PMMA bone cement. Experimental studies suggest that these approaches may help preserve PMMA polymerization and mechanical properties while facilitating more sustained local drug release. However, these findings remain at the proof-of-concept and preclinical stages, and their clinical efficacy, long-term safety, manufacturing scalability, sterilization stability, and translational feasibility have yet to be established. Therefore, such bioengineered delivery systems should currently be regarded as promising investigational strategies rather than established clinical solutions.

Future research should focus on several key areas: (1) clinical translation and rigorous validation of localized rifampin delivery systems; (2) clarification of the optimal timing and patient selection for rifampin initiation in DAIR; (3) identification and evaluation of effective companion antibiotics, particularly in the context of antimicrobial resistance; and (4) implementation of therapeutic drug monitoring and pharmacogenomic approaches to improve individualized rifampin therapy and mitigate systemic toxicity. Addressing these gaps may help define the most appropriate role of rifampin while facilitating the development of safer and more effective strategies for the management of biofilm-associated PJI.

## Supplementary Information

Supplementary Material 1.

## Data Availability

No datasets were generated or analysed during the current study.
