# Supplementary material for: The role, challenges, and innovations of rifampin in the management of periprosthetic joint infections: a narrative review
Source: Arthroplasty. 2026 Nov 3;8:83. doi: 10.1186/s42836-026-00445-z (PMC13729583; doi:10.1186/s42836-026-00445-z)
Supplement: Supplementary file 1 [file 42836_2026_445_MOESM1_ESM.docx]

**Supplementary material**

| **Database** | **Search strategy (August 9, 2026)** | **Number of articles** |
| --- | --- | --- |
| **Pubmed** | ((((rifampin[Title/Abstract]) AND (periprosthetic joint infection[Title/Abstract]))) OR (rifampicin[Title/Abstract])) AND (periprosthetic joint infection[Title/Abstract]) | 95 |
| **Web of Science** | rifampin (Abstract) and periprosthetic joint infection (Abstract) or rifampicin (Abstract) and periprosthetic joint infection (Abstract) | 111 |
| **Scopus** | (TITLE-ABS-KEY(periprosthetic joint infection) AND TITLE-ABS-KEY(rifampin) OR TITLE-ABS-KEY(periprosthetic joint infection) AND TITLE-ABS-KEY(rifampicin)) | 729 |
| **China National Knowledge Infrastructure** | （摘要：利福平(精确)）AND（摘要：置换(精确)）AND（摘要：感染(精确)） | 51 |
| **Wanfang, China Biology Medicine Disc** | 摘要:("利福平") and 摘要:("置换") and 摘要:("感染") | 65 |
| **China Science and Technology Journal (CSTD) databases** | 摘要=利福平 AND摘要=置换 AND摘要=感染 | 39 |
